# Dr. William G. Law

**Published:** 1920-06

**Authors:** 


### OBITUARY

Dr. William G. Law. Dr. Law died in New York City, January 30, 1920, of influenza. Dr. Law was a graduate of the University of Michigan, 1899. He located in Flint for practice, but soon went to Berlin, Germany, where he specialized in orthodontia. Here he built up a large and lucrative practice. Because of the war he moved to New York City and made a successful practice in a short time. He had planned to start a school of orthodontia in New York, but his illness interrupted this. He was a man of unusual energy and enthusiasm for his work. He made many friends and would undoubtedly have developed one of the large and successful practices of New York. His wife and daughter survive him. His numerous friends will mourn his death.

